# CD38, a Receptor with Multifunctional Activities: From Modulatory Functions on Regulatory Cell Subsets and Extracellular Vesicles, to a Target for Therapeutic Strategies

**DOI:** 10.3390/cells8121527

**Published:** 2019-11-27

**Authors:** Fabio Morandi, Irma Airoldi, Danilo Marimpietri, Cristiano Bracci, Angelo Corso Faini, Roberto Gramignoli

**Affiliations:** 1 Laboratory of Stem Cell and Cell Therapy, IRCCS Istituto Giannina Gaslini, 16147 Genova, Italy; irmaairoldi@gaslini.org (I.A.); danilomarimpietri@gaslini.org (D.M.); 2 Laboratory of Immunogenetics, Department of Medical Sciences, University of Torino, 10126 Torino, Italy; cristiano.bracci@edu.unito.it (C.B.); angelo.faini@edu.unito.it (A.C.F.); 3 CeRMS, University of Torino, 10126 Torino, Italy; 4 Department of Laboratory Medicine, Division of Pathology, Karolinska Institutet, SE-171 77 Stockholm, Sweden; roberto.gramignoli@ki.se

**Keywords:** CD38, regulatory cells, immune-modulation, adenosine

## Abstract

CD38 is a multifunctional cell surface protein endowed with receptor/enzymatic functions. The protein is generally expressed at low/intermediate levels on hematological tissues and some solid tumors, scoring the highest levels on plasma cells (PC) and PC-derived neoplasia. CD38 was originally described as a receptor expressed by activated cells, mainly T lymphocytes, wherein it also regulates cell adhesion and cooperates in signal transduction mediated by major receptor complexes. Furthermore, CD38 metabolizes extracellular NAD^+^, generating ADPR and cyclic ADPR. This ecto-enzyme controls extra-cellular nucleotide homeostasis and intra-cellular calcium fluxes, stressing its relevance in multiple physiopathological conditions (infection, tumorigenesis and aging). In clinics, CD38 was adopted as a cell activation marker and in the diagnostic/staging of leukemias. Quantitative surface CD38 expression by multiple myeloma (MM) cells was the basic criterion used for therapeutic application of anti-CD38 monoclonal antibodies (mAbs). Anti-CD38 mAbs-mediated PC depletion in autoimmunity and organ transplants is currently under investigation. This review analyzes different aspects of CD38’s role in regulatory cell populations and how these effects are obtained. Characterizing CD38 functional properties may widen the extension of therapeutic applications for anti-CD38 mAbs. The availability of therapeutic mAbs with different effects on CD38 enzymatic functions may be rapidly translated to immunotherapeutic strategies of cell immune defense.

## 1. Introduction

CD38 is a surface molecule, first identified as an activation marker of T lymphocytes [1], that plays an important role in the transduction of activating and proliferating signals [2] in a wide variety of immune cells. It is expressed early in the differentiation of CD34^+^ stem cells and remains in mature, but not resting, immune cells, including T cells, B cells, granulocytes and NK cells. It is composed of a single chain of 45 kDa, with three different domains that are (i) intracellular (20 amino acids), (ii) trans-membrane (23 amino acids) and (iii) extracellular (257 amino acids), with 2 to 4 N-linked oligosaccharide chains containing sialic acid residues [3]. However, the expression of CD38 is not confined to immune cells, either in mature cells or precursors, but this molecule is also expressed in solid tissues, such as the brain, eye, prostate, gut, pancreas, muscle, bone and kidney [4]. CD38 is also expressed at the maternal-fetal interface, where it has a role in the modulatory function of regulatory cells subsets that protect (semi)allogeneic fetal tissues from the maternal immune system [5]. The broad expression of CD38 is conceivably related to its protective effects that are exerted by multiple mechanisms, including (i) impediment in mitochondrial alterations; (ii) enhancement of energy metabolism; (iii) prevention of all forms of cell death including apoptosis, necrosis and autophagy; (iv) inhibition of inflammation; and (v) direct increase of anti-oxidation capacity in cells and tissues.

As mentioned, CD38 was initially described as a receptor molecule, that binds the specific ligand CD31, a surface molecule mainly expressed by endothelial cells [6]. It has been clearly documented that CD38 is able to establish strong lateral associations with professional signaling complexes of different cell lineages (i.e., CD3/TCR in T lymphocytes, BCR/CD19/CD21 in B lymphocytes and CD16/CD61 in NK cells). The CD38-containing complexes are mainly located in lipid rafts of the cell membrane. Thus, CD38 has a critical role in transducing activating signals in immune cell populations [4].

In juxtaposition to the definition of CD38 as an activation marker, was the finding that plasma cells, terminally differentiated elements of the B lineage, express the highest levels of surface CD38. Although the role of CD38 during plasma cell differentiation is still unclear, it is well established that it functions also as an adhesion molecule endowed with the ability to adhere to heterotypic cells, to transduce activating signals involved in the control of Ca^2+^, and to act as an ecto-enzyme.

CD38 may exist in a soluble form; thus, being present in biological fluid at different levels during physiological and pathological conditions [7]. The first evidence highlighting the ecto-enzymatic activity of CD38 was provided by the work of Lee and coworkers [8]. They documented that membranes purified from the surface of human erythrocytes were endowed by a hydrolase enzymatic activity and were able to convert NAD^+^ to cyclic ADP-ribose (cADPR), and subsequently, to ADP-ribose. A few months later, the same group pinpointed that such enzymatic activities were related to the CD38 molecule [9]. These findings were further sustained by the observation that retinoic acid was able to induce concomitantly NAD^+^ hydrolase activity and up-regulation of CD38 expression in HL60 cells, since NAD^+^ hydrolase could be immune-precipitated using an anti-CD38 antibody. In addition, CD38 cDNA transfer into *Escherichia Coli* conferred a NAD^+^ hydrolase activity to engineered cells [10]. However, the unambiguous demonstration that the CD38 molecule was endowed with enzymatic functions was reported by Howard and coworkers, using a synthetic cDNA encoding the extracellular domain of CD38 molecule, which encoded a soluble CD38 molecule. Such molecule, in the presence of NAD^+^, produced and hydrolyzed cADPR, and the latter molecule was able to induce B cell proliferation, underlying a possible role of CD38 in lymphocyte activation and function [11].

Recently, several studies reported CD38 as a part of ecto-enzymatic networks that generate adenosine (ADO) from different substrates, including ATP and NAD^+^. The “canonical” pathway for ADO production is composed of CD39 (NTP diphosphohydrolase) that converts ATP to ADP and AMP, and CD73 (ecto-5′-nucleotidase) that converts AMP to ADO [12]. CD39 and CD73 are both commonly expressed by regulatory T cells (Treg) and play an important role in Treg-mediated immune-modulatory functions [13]. In this context, Peola and coworkers firstly demonstrated that CD38 ligation by monoclonal antibodies (mAbs) induced the export of pre-formed CD73 from an intracellular pool to the cell surface [14]. Next, a functional link between CD38 and CD73 was clearly documented by Horenstein and coworkers [15], who envisaged a novel enzymatic pathway for ADO production. The novel “alternative” axis is initiated by CD38 converting NAD^+^ to cADPR, further metabolized by ecto-nucleotide pyrophosphatase phosphodiesterase 1 (NPP1, also known as CD203a or PC-1) that generates AMP, which subsequently converted to ADO by the enzymatic activity of CD73. Intriguingly, this pathway is also functional in a discontinuous way, where each ecto-enzyme is expressed by different cell subsets nearly located in a closed microenvironment [16].

Such findings established that CD38 is much more than an activating receptor, since it is involved in the regulatory functions of several immune and non-immune cell populations through the generation of ADO; thus, representing a key molecule of an immune-modulatory pathway.

## 2. Immune-Modulatory Role of CD38 in T Lymphocytes: Implication for Treg Activities

Several studies have described the role of CD38 as an immune-modulatory molecule in T cell subsets with regulatory properties. The first evidence came from the work of Read and coworkers [17], who have identified among murine CD45RB^low^ memory CD4^+^ T cells, a CD38^neg^ cell subpopulation containing conventional memory T cells able to proliferate and produce cytokines in response to recall antigens. Conversely, CD38^+^ T lymphocytes suppress the proliferation of CD38^−^ T cells, although in the absence of IL-10/TGF-β secretion. This concept has been reinforced by Martins and coworkers [18], demonstrating that CD45RB^low^CD38^+^ T cells play an immune-modulatory role by inducing anergy in self-reactive T lymphocytes in vivo in NOD mice; thus, protecting animals from diabetes.

Afterwards, Bahri and coworkers identified a specific subset of regulatory CD8^+^ T cells that express high levels of CD38 on their surface and are present in both mice and humans. Such T cell subset, that is, CD38^hi^CD8^+^, is capable of suppressing CD4^+^ T lymphocytes’ proliferation and of mitigating the symptoms of experimental autoimmune encephalomyelitis in vivo in pre-clinical models. The additional finding that CD8^+^ T lymphocytes not expressing CD38 are avoided by such activity, clearly demonstrated that CD38 is involved in the modulatory functions of regulatory T cells [19]. Subsequently, Chen et al. reported that in the absence of CD38, NOD mice (CD38 knock-out mice) developed accelerated autoimmune diabetes and impaired regulatory T cell development [20].

More recently, dendritic cells exposed in vitro to BPZE1 pertussis vaccine have been shown to be capable of generating unconventional CD4^+^/CD8^+^ regulatory T cells characterized by high levels of ecto-enzymes belonging to both canonical (CD39/CD73) and non-canonical (CD38/CD203a/CD73) adenosinergic pathways. Such cells are able to produce ADO starting from ATP and NAD^+^. Experiments performed using specific inhibitors of CD38, CD39 and CD73 clearly demonstrated that both pathways are crucial for CD4^+^/CD8^+^ regulatory T cell functions, which are strictly related to the resulting levels of ADO [21].

The immune-modulatory role of CD38 on classical CD4^+^CD25^+^FoxP3^+^ regulatory T cells (Tregs) has been described by Patton and colleagues, measuring high levels of CD38 in a subset of Tregs originating in the thymus, and in splenic Tregs. CD38^hi^ Tregs display a superior modulatory activity compared to CD38^−^ counterparts. The latter cells are not characterized by up-regulated CD73 expression on their surface, further confirming the role of CD38/CD203a/CD73 adenosinergic pathway in the modulatory functions of Tregs [22]. Recently, a subset of CD69^+^ Tregs have proved a robust modulatory activity compared to CD69^−^ counterparts, and such function was related to high levels of immune-modulatory molecules, including CD38 [23]. A different and functional alternative pathway for immune-suppression driven by CD38 has been reported by the interesting findings from the study of Kim and coworkers, who have demonstrated that soluble (s)CD38 released by seminal vesicles was capable of triggering an immune-modulatory microenvironment at the maternal/fetal interface by inducing the generation of Tregs and tolerogenic dendritic cells. Thus, CD38 not only is directly involved in modulatory functions of Tregs, but it may also induce Treg generation [24]. The unambiguous proof of the crucial role of CD38 in the modulatory functions of Tregs may be found in recent clinical studies carried out using anti-CD38 therapeutic antibodies. Daratumumab is a human mAb against CD38, currently tested on patients with multiple myeloma (MM) and other hematological malignancies [25,26]. Daratumumab targets and eliminates CD38^+^ malignant cells through different mechanisms (complement-dependent cytotoxicity, antibody-dependent cytotoxicity and antibody-dependent phagocytosis). Cells from peripheral blood and bone marrow (BM) of patients undergoing daratumumab monotherapy have been analyzed before and during therapy, and at relapse. It has been shown how chemotherapeutic treatment erases CD38^+^ Tregs (which are more suppressive in vitro than their CD38^−^ counterparts). Moreover, helper and cytotoxic T cells resulted significantly increased in treated patients, in terms of absolute counts and CD8^+^:CD4^+^, CD8^+^:Treg and memory:naïve ratios. Such effects are even more evident in patients with partial or good responses to daratumumab treatment, suggesting that depletion of Tregs is a crucial additional mechanism of action of the mAb [27]. Feng and coworkers have obtained similar data, although studying a different aspect; that is, the effects of isatuximab (a chimeric mouse/human monoclonal anti-CD38 antibody developed by ImmunoGen and Sanofi-Aventis and licensed under the name of SAR-650984) administered with pomalidomide/lenalidomide on peripheral blood mononuclear cells (PBMNCs) from MM patients and normal donors. In that multicentric study, they observed a higher percentage of CD38^hi^ Tregs in PBMNC from myeloma patients than in controls. In vitro analysis confirmed that CD38 expression and the percentage of CD38^hi^ Treg were increased in PBMNCs by pomalidomide/lenalidomide, whereas isatuximab induced a depletion of CD38^+^ Tregs, that was paralleled by a restored proliferation and function of effector T cells. Accordingly, isatuximab treatment enhanced lysis of myeloma cells performed by cytotoxic T cells and NK cells in vitro. In addition, they have demonstrated that co-culture of PBMNC with myeloma cells induced CD38^hi^ Treg, and that treatment of PBMNC with isatuximab decreased the percentage of induced Tregs [28]. Collectively, these studies pinpointed that CD38 is crucial for in vivo Treg modulatory functions and generation, and confirmed CD38 targeting approach by mAbs as efficient treatment to deplete CD38^hi^ Tregs and revert tumor-induced immune-modulation.

## 3. CD38 and Regulatory B Cells

IL-10-producing regulatory B cells (Bregs) have been identified many years ago in mice as lymphocytes involved in down-regulation of inflammation, making them potentially important for the maintenance of tolerance. Several preclinical studies have also addressed the role of different subsets of Bregs in mice, using different surface markers, such as (i) CD5 alone [29] or in combination with CD19 and CD1d [30], and (ii) CD19, CD21, CD23 and CD24 in combination with CD1d [31]. Last year, Burlock B et al. asked the question of whether CD38 may play a role in the expression and function of regulatory B cells (also known as B10 cells) using, as experimental model, *Cd38*^−/−^ mice developing a milder pristane-induced lupus disease [32]. These experiments revealed that in pristane-treated mice, the frequency of spleen CD19^+^CD1d^hi^CD5^+^ B cells, which are highly enriched in B10 cells, was significantly increased in *Cd38*^−/−^ splenocytes compared to wild type, while the frequency of peritoneal plasmacytoid dendritic cells, which are major type I interferon producers, was greatly diminished. Functional ex-vivo assays showed increased frequencies of B10 cells in *CD38*^−/−^ murine splenocytes, compared to wild type mice, upon stimulation with an agonist anti-CD40 mAb. Thus, those results strongly suggested that *Cd38*^−/−^ mice are better suited than wild type mice to generate and expand regulatory B10 cells, following the appropriate stimulation [32].

Several recent works have also identified IL-10 producing regulatory B cells in humans and have begun to unravel their phenotype and mode of suppression. The cell surface phenotype of human Bregs includes CD38, CD27, CD24 and CD5. Mechanisms of suppression may imply inhibition of CD4^+^ T proliferation, inhibition of Th1 differentiation, induction of regulatory T cells and suppression of monocyte activation [33]. Additional studies reported that B cells with phenotypic features of CD19^+^CD24^hi^CD38^low^ and CD19^+^CD24^hi^CD38^−^ cells do not play any modulatory activity in vitro; thus, indicating that CD38 is a key molecule involved in the typical immune-suppression operated by Breg cells, based on the following considerations. The modulatory functions of Bregs are, at least in part, dependent on IL-10 secretion and CD80/CD86 engagement [34], but since high expression of CD38 is a peculiar feature of these cells, it is conceivable that the enzymatic function of CD38 associated with ADO production may be involved in their modulatory activity. That hypothesis was partially confirmed by Figueirò and coworkers, who have demonstrated that B cells activated in vitro showed a higher percentage of CD39^hi^ cells. Interestingly, CD38^hi^ cells were present only in the CD39^hi^ cell fraction, suggesting that the latter cells are at least in part composed by CD19^+^CD24^hi^CD38^hi^ Bregs. These cells also expressed CD73 and were able to produce 5′-AMP and ADO from ATP. Such production was only partially inhibited by anti-CD39 blocking antibodies, suggesting that both the canonical and alternative pathways may be functional [35]. Another study has addressed the crucial role of CD38 for the modulatory functions of Bregs in pre-clinical models. *CD38*^−/−^ mice develop autoimmunity, displaying high levels of anti-nuclear antibodies, anti-double strand DNA auto-antibodies and a reduced survival, and such effects have been related to a defective function of Bregs [36].

Several reports described the role of Bregs in the pathogenesis of different human diseases, including thrombocytopenia [37], chronic hepatitis B virus infection [38], rheumatoid arthritis [39] and chronic graft-versus-host disease [40]. The mechanism of action is related to the inhibition of T cell response and/or to the expansion of Tregs. Conversely, Breg function is defective in patients affected by common variable immunodeficiency [41], psoriasis [42], lupus erythematosus [43] and systemic sclerosis [44].

Breg modulatory function may be enhanced in cancer patients, wherefore malignant cells may promote survival and proliferation of Breg, which may support an immune-privileged microenvironment by dampening anti-tumor immune response [45]. Circulating Bregs can also be expanded in patients with solid and hematological tumors [46]; hence, contributing to Tregs’ expansion [47]. Accordingly, mAbs capable of targeting and eliminating not only tumor cells but also Bregs may boost therapeutic effectiveness. As previously mentioned, daratumumab may target CD38^+^ tumor cells but also CD38^hi^ Tregs. A recent study showed a complete depletion of Bregs in daratumumab-treated patients. The number of Bregs did not recover and remained very low during the treatment [27].

## 4. Immune-Regulatory Functions of CD38 on Innate Immunity: Focus on CD16^−^CD56^bright^ NK Cells

Few studies have delineated an immune-regulatory role for the CD16^−^CD56^+^ NK cell subset in autoimmune disorders [48,49,50,51]. The critical role of IL-10 and granzyme K has been described [52,53]. Conversely, we described an additional mechanism underlying the immune-regulatory properties of CD16^−^CD56^bright^ NK cells, where CD38 plays a critical role [54]. Firstly, starting from a deep analysis of the expression of ecto-enzymes belonging to classical (CD39, CD73) and alternative (CD38, CD203a, CD73) adenosinergic pathways in CD16^+^CD56^dim^ and CD16^−^CD56^bright^ NK cells, we highlighted that the latter cells express higher levels of CD39 and CD73. More importantly, CD203a is exclusively expressed by CD16^−^CD56^bright^ NK cells; thus, suggesting that the alternative adenosinergic pathway is operating only in this subset of NK cells. Accordingly, CD16^−^CD56^bright^ NK cell subset produces ADO from different substrates; in particular, starting from NAD^+^ (substrate of CD38) and abrogating the proliferation of autologous activated CD4^+^ T cells at different NK: T cell ratios. Furthermore, such an inhibitory effect was partially reverted using exclusively kuromanin, a specific inhibitor of CD38, confirming its central role for NK cell-mediated immune-modulation [54]. Supportive evidence comes from the analysis of the synovial fluid from patients affected by juvenile idiopathic arthritis, an autoimmune condition characterized by sustained inflammation of joints. CD16^−^CD56^bright^ NK cells isolated from synovial fluid exhibited a reduced CD38 and CD73 expression, compared to peripheral blood counterparts. Accordingly, such NK cell subset revealed a limited modulatory function and delayed production of ADO in the presence of different substrates compared to peripheral blood cells. These data demonstrated that the regulatory function of CD16^−^CD56^bright^ NK cells may be dampened in autoimmune/inflammatory diseases, through a down-regulation of CD38 expression and function [54].

An indirect observation of CD38 role in the modulatory functions of CD56^bright^ NK cells also arises from the work performed by de Jonge and coworkers, who reported that a high percentage of the latter cells in the peripheral blood negatively affected overall survival of stage IV melanoma patients. Interestingly, CD56^bright^ NK cells from the same patients displayed a higher CD38 expression, paralleled by a lower TNF-α and GMCSF production in vitro, suggesting an increased immune-regulatory function of patients’ CD56^bright^ NK cells. Thus, the gain of the modulatory function of the latter cells in vivo was related to an increased expression of CD38 on their surface [55].

## 5. CD38 Expression and Function in Non-Hemopoietic Immunoregulatory Cells

CD38 and other components of adenosinergic pathways have been described on non-hematopoietic immune-regulatory cells. Constitutive or inducible expression of canonical and alternative ectonucleotidases in different types of regulatory cells have been ascribed to their role in modulating inflammatory response and immune-recognition.

Mesenchymal stromal cells (MSCs) have been extensively characterized in the last decade for their immune-regulatory properties in different experimental and in vivo conditions. MSCs regulate many effector functions of innate immune cells, such as dendritic and NK cells, and inhibit T and B cell functions and proliferation through different mechanisms. Based on extensive clinical infusions in allogenic settings where the immune response was not generated, contrariwise modulated, MSCs have been reported to express and secrete several immune-modulatory molecules, including prostaglandin (PG) E_2_, indolamine dioxigenase (IDO) and HLA-G, that interact with specific inhibitory receptors on immune effector cells, leading to the abrogation of their functions [56]. Characteristic expression of CD73 has become typecasting for the identification of MSC of various origins, for more than a decade [57]. CD73, together with CD39 expression and related ADO production by MSC have been proven to generate inhibitory effects on T lymphocytes by short-circuiting with adenosine receptors expressed on the latter cells [58]. In addition, our group has demonstrated that CD38 and CD203a (belonging to the “alternative” pathway) are also present on BM-derived MSCs, and that CD38 expression is downregulated during osteoblast differentiation. Thus, MSCs are potentially equipped with both complete adenosinergic pathways. However, the alternative pathway started by CD38 is fully functional only in undifferentiated MSCs, which are likely able to generate high amounts of ADO; that is related to their modulatory functions [16].

Interesting findings also come from studies regarding myeloid-derived suppressor cells (MDSCs), a heterogeneous population of immature myeloid cells characterized by high plasticity and immune-modulatory abilities [59]. MDSCs are able to inhibit T and NK cell functions through the (i) depletion of extracellular nutrients, such as L-Arginine and NAD^+^; (ii) production of immune-modulatory molecules, such as IL-10, TGF-β, adenosine and PGE_2_; and (iii) induction of expression of immune checkpoint inhibitors, such as programmed death ligand (PDL)-1 [59]. In one such a scenario, CD38 seems to play a dual modulatory function: on one hand, CD38 is involved in the degradation of NAD^+^, due to its ability to deplete this molecule in the extracellular compartment. On the other hand, CD38 is the lead molecule of alternative adenosinergic pathway, leading to the generation of the immune-modulatory partner ADO. In this context, Li and colleagues have demonstrated that ADO is generated by the classical CD39/CD73 adenosinergic pathway, and that ovarian cancer patients treated with metformin display an increased anti-tumor activity paralleled by a reduction of circulating CD39^+^/CD73^+^ MDSCs [60]. However, the contribution of the alternative adenosinergic pathway initiated by CD38 cannot be ruled out. Nevertheless, the crucial role of CD38 expression on MDSCs has been also demonstrated by the work by Karakasheva and coworkers, where superior modulatory activity in CD38^hi^ MDSCs compared to CD38^low^ counterparts has been measured. This finding was associated with an increased number of CD38^hi^ MDSCs in the peripheral blood of cancer patients in advanced stage, reinforcing the concept that the latter cells are the major players involved in suppression of anti-tumor immune response [61]. Finally, supportive evidence on the critical role of CD38^+^ cells has been shown in anti-CD38 daratumumab treatments, where anti-cancer activity has been primarily related to the depletion of CD38^+^ MDSCs [27].

Human amnion epithelial cells (hAECs) represent a novel regulatory cell subset that has been recently translated in clinical settings [62,63,64]. These fetal-derived stem cells display several immune-modulatory properties that are mediated by surface molecules involved in cell-to-cell interactions (such as HLA-G or canonical ecto-nucleotidases) or by secreted mediators (IL-10, TGF-β1, PGE2 and IDO) [65]. The secretion of such molecules, together with the enhanced expression of non-polymorphic HLA-G, have been described to increase upon exposure to pro-inflammatory molecules, such as INF-γ and IL-1β [66]. Moreover, hAECs are able to inhibit chemotaxis of macrophage and neutrophils and to suppress proliferation of T and NK cells. The mechanism(s) involved in such inhibition are largely unknown, with preliminary support in the expression of mRNA for TNF-α, Fas ligand (FasL), tumor necrosis factor-related apoptosis-inducing ligand (TRAIL), TGF-β and macrophage migration-inhibitory factor (MIF) [67]. To further address such issue, our group performed a study to evaluate the expression and function of canonical and alternative adenosinergic pathways in hAECs. We have recently shown that hAECs constitutively express complete and functional canonical (CD39/CD73) and alternative (CD38/CD203a/CD73) adenosinergic pathways, leading them to generate ADO starting from NAD, AMP and ATP. In that seminal study, hAECs were proven able to suppress T-cell proliferation in vitro through a mechanism, at least in part, related to ADO production (as witnessed by the recovery of T cell proliferation when specific inhibitors of adenosinergic ecto-enzymes were provided). Moreover, ADO may be responsible for the expansion of regulatory T and B cells induced by co-culture of hAEC with PBMNC from normal donors [68]. Thus, we delineated a novel role for CD38 in the context of hAEC-mediated modulation of immune response; that needs to be taken into account in the view of clinical applications for the latter cells.

## 6. CD38 Expression on Extracellular Vesicles: Another Mechanism of Immune-Modulation?

Extracellular vesicles (EV) have been attracting interest and have been extensively studied in the last few years, since they might represent an adjunct to cell transplant approaches or standard pharmacological treatment. Moreover, higher levels of circulating EVs have been reported in cancer patients than in healthy controls. Such EVs were characterized by the expression of specific markers of the original tumor cells, suggesting that such vesicles may constitute a cellular strategy performed by tumor cells to overcome the recognition by the immune system [69].

Our group has recently carried out two different sets of studies aimed to characterize the expression and function of CD38, along with other ecto-enzymes, on EVs isolated from patients with MM [70] and neuroblastoma (NB) [71].

We successfully proved the presence of complete adenosinergic machinery (CD38, CD203a, CD39 and CD73) on EV isolated from both groups of subjects. In the myeloma setting, EVs isolated from BM of MM patients are characterized by higher percentages of CD38^hi^, CD230a^hi^, CD73^hi^ and CD39^hi^ EVs than those from controls (represented by patients with smoldering myeloma or monoclonal gammopathy of undetermined significance). Accordingly, such EVs were more effective in the production of ADO from ATP, AMP and NAD^+^ than those isolated from controls, confirming that both adenosinergic pathways are functional. Collectively, these preliminary results suggest that the release of EVs by cancerogenic cells, endowed with both adenosinergic machineries capable of generating ADO from different substrates, may represent an efficient immune escape mechanism performed by malignant cells to circumvent immune response and to promote neoplastic growth in the BM microenvironment. Such conclusion was supported by the direct correlation measured between the percentage of CD38^hi^ EV and malignant plasma cells in the BM [70]. Notably, the presence of CD38 and CD39 on EV isolated from BM of MM patients has been recently confirmed by electron microscopy analysis [72].

Similar results were obtained analyzing EVs from NB patients [71]. Constitutive high levels of CD38, CD203a, CD39 and CD73 expression have been described (superior to those from healthy controls) in terms of (i) percentage of positive EVs, and (ii) levels of expression, witnessed by a higher fluorescence intensity. Moreover, NB patient-derived EVs exhibited a higher percentage of CD38^hi^ and CD203a^hi^ EVs than healthy controls, confirming the pivotal role of CD38 in such setting. Indeed, the percentage of CD38^hi^ and CD203a^hi^ EVs correlated with degree of BM infiltration by malignant NB cells. Likely, EVs derived from NB patients were characterized by a massive production of ADO generated by ATP, NAD, ADPR and AMP metabolism, confirming that both pathways are functional. Moreover, enzymatic activity detected in NB patients’ EVs was higher than that detected in that isolated from healthy controls. Furthermore, such NB-derived EVs proved to efficiently inhibit T cell proliferation in vitro, confirming their modulatory functions.

Interestingly, our study also described as CD38^+^ and CD73^+^ EVs in the BM negatively affected the event-free survival of NB patients, confirming their critical impact on the immune-privileged microenvironment of neoplastic cells, that, in turn, support the metastatic spread of NB cells [71].

The constitutive presence of surface CD38 has also been described on another setting of tumor-derived exosomes. Zumaquero and coworkers reported how exosomes released by lymphoblastoid cell lines are CD38^+^ and highly functional in terms of enzymatic activity, as witnessed by a potent GDP ribosyl cyclase activity in vitro. Additional evidence supporting CD38 presence on such exosomes arises from immunoprecipitation assays, which revealed that CD38 is associated with signaling complexes containing the tetraspanin CD81, Hsc70 and the tyrosine kinase Lyn [73].

**Figure 1:**
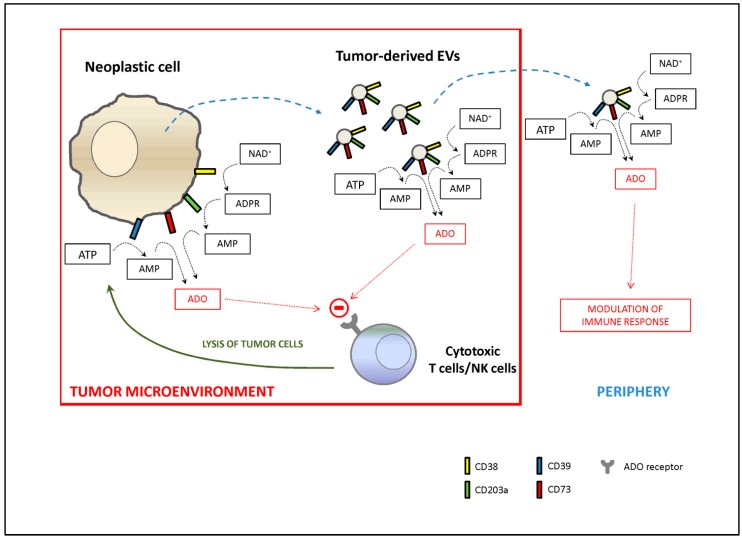
Generation and function of tumor-derived extracellular vesicles (EVs). CD38, CD39, CD203a and CD73 molecules are commonly expressed by several solid and hematological tumors. In addition, tumor cells are able to release EVs that may be endowed with the same adenosinergic ecto-enzymes. As result, both tumor cells and tumor-derived EVs are capable of generating of adenosine (ADO) starting from ATP (through the action of CD39 and CD73) or NAD^+^ (through the action of CD38, CD203a and CD73). ADO produced in the tumor microenvironment is able to interact with ADO receptors on T lymphocytes and NK cells, shutting down anti-tumor immune response. Moreover, tumor-derived EVs, upon distribution by circulatory stream, may vehicle peripheric effect, modulating immune response by cells expressing ADO receptors (T and B lymphocytes, NK cells and monocytes/macrophages).

In conclusion, the authors of afore studies concurred that functional vesicles are directly released by neoplastic cells and/or induced in resident immune cells in response to soluble factors released by malignant cells, to confer a growth advantage to cancer, as described in Figure 1.

## 7. Therapeutic Approaches Based on Targeting CD38

CD38 is a cell surface molecule with multiple and different functions. This molecule has been initially described as primarily involved in cell adhesion and signaling in leukocytes, acting as a receptor through the interaction with specific ligands. Later, CD38’s role as ecto-enzyme in NAD^+^ degradation made it a critical player in adenosinergic pathways leading to ADO generation [74].

We have here reviewed the principal studies that describe the CD38 enzymatic function, which is crucial for the immune-modulatory activities accomplished by lymphoid and non-lymphoid regulatory cell subsets. Based on these observations, and considering that CD38 is highly expressed especially in mature cells, but only marginally in lymphoid progenitors [75], the latter molecule represents an optimal target for the development of immunotherapeutic strategies. The behind logic is evident and relatively simple: a target molecule for immunotherapy should be selected taking into account its high expression on tumor cells and the cytotoxic effect of the corresponding antibody. For this reason, the most suitable tumor used for CD38 targeted therapies is the MM, still considered incurable. Two major therapeutic anti-CD38 mAbs have been developed: daratumumab and isatuximab [76].

Daratumumab is a fully human mAb that was initially approved in 2015 for patients having failed at least three prior therapies. The high effectiveness of daratumumab, in combination with dexamethasone and bortezomib or lenalidomide, in relapsed/refractory MM patients has been clearly established by network meta-analysis studies of control clinical trials [77]. However, clinical trials with daratumumab as monotherapy in the same type of patients have demonstrated the drugs’ efficacies, witnessed by high objective response rate and improved progression free survival. Although adverse effects have been shown and include neutropenia and thrombocytopenia, daratumumab combinations as frontline therapy in newly diagnosed MM patients have been approved. Daratumumab is also currently employed for the treatment of patients with immunological disorders, including amyloid light-chain amyloidosis [78], refractory red cell aplasia [79], primary effusion lymphoma [80] and refractory autoimmune hemolytic anemia [81]. Treatment was safe, well tolerated and displayed good responses. Furthermore, since autoantibody levels are reduced in daratumumab treated MM patients, this antibody is currently under evaluation for the treatment of patients with autoantibody-dependent autoimmune disorders [82]. Daratumumab’s therapeutic effectiveness of has also been tested in preclinical models of T-cell acute lymphoblastic leukemia, with promising results [83].

Isatuximab is a chimeric mouse/human mAb with significant differences from daratumumab in terms of mechanisms of action. Its anti-tumor efficacy appears to be primarily related to antibody-dependent cellular cytotoxicity (ADCC) and inhibition of ecto-enzyme activity. Starting from data derived from preclinical studies reporting that isatuximab’s killing activity was greatly increased when combined with an immune-modulator (e.g., pomalidomide or dexamethasone) [84], clinical trials have been implemented using isatuximab alone or in combination with carfilzomib, bortezomib, lenalidomide, dexamethasone and pomalidomide. Phase 1b studies with isatuximab in combination with pomalidomide/dexamethasone revealed that such a formulation was well tolerated and elicited partial responses associated with manageable toxicity in 62% of relapsed/refractory MM patients [85,86].

**Figure 2:**
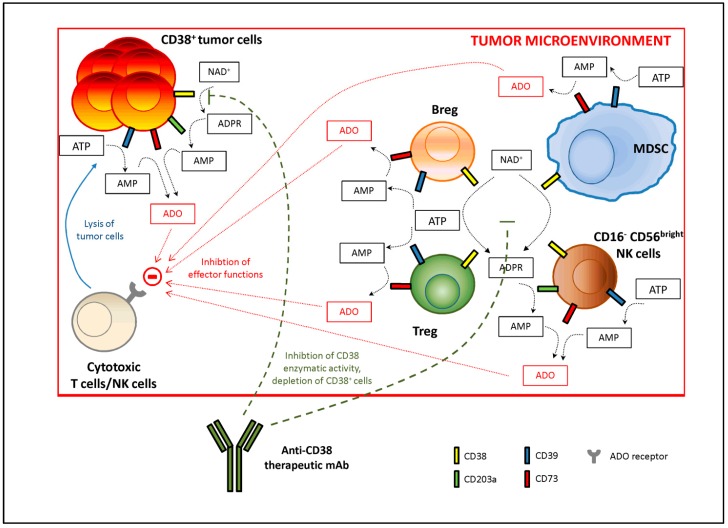
Immune-modulatory tumor microenvironment and mechanism(s) of action of therapeutic anti-CD38 monoclonal antibodies (mAbs). Tumor cells are able to attract within the tumor microenvironment different regulatory subsets, such as regulator T cells (Tregs), regulatory B cells (Bregs), CD16^−^CD56^bright^ NK cells and myeloid-derived suppressor cells (MDSC). All these cells may co-operate in the production of adenosine (ADO) starting from ATP or NAD^+^. CD38^+^ tumor cells are able to generate ADO themselves. ADO produced in the tumor microenvironment is able to interact with ADO receptors on T lymphocytes and NK cells, shutting down anti-tumor immune response. Immunotherapies using anti-CD38 mAbs can overcome this immune suppression by (i) blocking CD39 enzymatic activity and (ii) targeting CD38^+^ regulatory cells, that are eliminated through antibody-dependent and complement-dependent cytotoxicity or antibody-dependent phagocytosis. This mechanism(s) drives to the elimination of CD38^+^ tumor cells, leading to an increased therapeutic response.

From a mechanistic point of view, mAbs that target CD38 have been described to efficiently erase not only CD38^+^ tumor cells, but also different populations of CD38^+^ regulatory cells, which are endowed with the highest immune-modulatory properties and with protective role to such invasive malignant cells against immune system recognition. Thus, the anti-tumoral therapeutic effect of these antibodies may be greatly enhanced. Figure 2 describes the immune-modulatory tumor microenvironment and the mechanism(s) of action of anti-CD38 antibodies.

However, some concerns have been recently raised by the finding that, in a myeloma setting, anti-CD38 ligation by specific antibodies on malignant plasma cells leads to the aggregation, polarization and release of EVs derived from cell membrane. Furthermore, treatment of patients with anti-CD38 antibodies is followed in vivo by a strong release of EVs at the tumor site, where they interact with infiltrating cell populations. Alternatively, EVs may be released in the periphery, where they interact with cells expressing Fc receptors (through anti-CD38 antibody that is still bound to surface CD38) such as monocytes and NK cells. More importantly, these EVs express adenosinergic molecules (CD39, CD203a, CD73) clustered in lipid domains; thus, endowing them with immune-modulatory properties [87].

## 8. Resistance to CD38 Antibodies: A Rationale for Multidrug Therapies

The mechanisms underlie the clinical benefits of CD38 antibodies, are mainly related to Fc-dependent immune effector mechanisms, such as ADCC, complement-dependent cytotoxicity (CDC) and antibody-dependent cellular phagocytosis (ADCP) together with the inhibition of ecto-enzymatic function and direct apoptosis induction. In addition, CD38 antibodies potentiate the host anti-tumor immunity through physical elimination of Tregs and Bregs, as well as MDSCs that typically exert immune-suppressive functions.

However, patients treated with CD38 antibodies are subjected to drug resistance that may occur with different mechanisms, as revealed by preclinical and clinical studies that evaluated the daratumumab effects. First, during daratumumab treatment it has been reported a rapid decrease in CD38 expression levels not only on tumor (e.g., MM) cell surfaces, but also in normal B, T and NK cells. Although such down-regulation is transient and observed in patients with sustained high quality responses as well, it is clear that such feature is associated with protection against ADCC and CDC [88] as well as reduction in target cell killing, due to the intrinsic ability of CD38 antibodies to preferentially bind cells with high levels of CD38. The CD38 decreased expression driven by daratumumab treatment may be related at least to the following additional mechanisms: (i) clustering of CD38 molecules into distinct polar aggregates, which can subsequently be released as tumor-derived microvesicles [89]; (ii) direct internalization of CD38; and (iii) active transfer of CD38-daratumumab complexes from tumor cell membrane to monocytes and granulocytes [90].

It is currently unknown whether tumor-associated factors, including mutations in the antigen processing and presentation pathways, loss of antigen expression or insensitivity to T cell effector molecules are associated with resistance to CD38-targeting antibodies. However, it has been reported that compensatory up-regulation of multiple inhibitory immune checkpoints, implicated in the resistance to PD-1 or PD-L1 inhibitors, may also contribute to development of resistance to CD38 antibodies.

All these mechanisms have to be taken in mind to design enhanced CD38-targeting antibody therapies. Significant levels of improvement can be achieved by adding agents capable of enhancing complement activation, NK-cell-mediated ADCC, macrophage-mediated ADCP and/or host-anti-tumor T cell immunity. Although a better understanding of mechanisms that contribute to resistance should be useful, several clinical trials are currently evaluating whether patients that develop resistance to a CD38 antibody may benefit from adding other drugs reversing resistance to CD38 antibodies.

CD38 expression on tumor cells may predict clinical response to daratumumab therapy. Indeed, patients who achieved at least a partial response to therapy displayed a higher CD38 expression on MM cells than unresponsive patients [88]. In this context, all-trans retinoic acid (ATRA) treatment is able to increase CD38 expression levels and reduce expression of the complement-inhibitory proteins CD55 and CD59 in MM cells. Furthermore, ATRA enhances the activity of daratumumab in vitro and in preclinical MM models. Thus, this study provided a rationale motivating the daratumumab/ATRA combined therapy for MM patients [91].

Immune-modulatory drugs (IMiD), such as thalidomide, lenalidomide and pomalidomide, are capable of triggering the degradation of the transcription factors Ikaros and Aiolos, which repress the expression of interferon-stimulated genes, including CD38. Thus, treatment with IMiD leads to the up-regulation of CD38 on MM cells, priming them to the daratumumab-induced NK cell-mediated ADCC [92,93]. A retrospective analysis of MM patients treated with daratumumab and pomalidomide confirmed that this combined therapy leads to a better clinical outcome, in terms of overall response rate and progression-free survival [94].

Similar effects may be achieved using DNA methyltransferases (DNMT), due to the following considerations. First, DNA methylation patterns change during MM progression, and clinically aggressive subtypes display DNA hypermethylation [95]. Second, hypermethylation of 195 genes, including *CD38* gene, correlated with a worse prognosis in MM patients [96]. Choudhry and coworkers identified a CpG island in the exon 1 of *CD38* gene and demonstrated that two DNMT, azacytidine and decitabine, up-regulate CD38 on MM cells, both at mRNA level and surface protein. Accordingly, in vitro ADCC was higher in DNMT-treated than in untreated cells, supporting the concept that DNMT may be used to improve daratumumab therapeutic efficacy [97].

Finally, another approach consists of administering different CD38 antibodies, characterized by different modes of action, when refractoriness to a specific CD38 Ab treatment has been experienced. However, although functional differences exist among CD38-targeting antibodies, it is currently unclear whether resistance to one may result in defiance to any other CD38 antibodies.

## 9. Conclusions

The enzymatic activity of CD38 (and related adenosinergic ecto-enzymes) plays crucial roles in regulating adaptive and innate immune response, both in physiological and pathological conditions. Such a functional axis may drive novel and more effective immunotherapeutic strategies, resulting in adjuncts to standard pharmacological treatments or cell transplant approaches for oncological treatments and regenerative medicine. Growing evidence on host and tumor-related features that drive resistance toward CD38 antibodies underlies differential therapeutic efficacy, and may lead to further optimization and individualization of oncological treatment for a better outcome in patients affected by different tumors and MM in particular.
